# Protective effect of coenzyme Q10 in cyclophosphamide-induced kidney damage in rats

**DOI:** 10.1590/1806-9282.20230990

**Published:** 2024-05-03

**Authors:** Ozlem Kara

**Affiliations:** 1 Kirsehir Ahi Evran University, School of Medicine, Department of Histology and Embryology – Kırşehir, Turkey.

**Keywords:** Cyclophosphamide, Coenzyme q10, Rat, Kidney, Toxicity

## Abstract

**OBJECTIVE:**

We aimed to investigate the effect of coenzyme q10 on cyclophosphamide-induced kidney damage in rats.

**METHODS:**

A total of 30 female Wistar-Albino rats were utilized to form three groups. In group 1 (control group) (n=10), no drugs were given. In group 2 (cyclophosphamide group) (n=10), 30 mg/kg intraperitoneal cyclophosphamide was administered for 7 days. In group 3 (cyclophosphamide+coenzyme q10 group) (n=10), 30 mg/kg cyclophosphamide and 10 mg/kg coenzyme q10 were given for 7 days via intraperitoneal route. Right kidneys were removed in all groups. Blood malondialdehyde levels and activities of catalase and superoxide dismutase were measured. Histopathological damage was evaluated by examining the slides prepared from kidney tissue using a light microscope.

**RESULTS:**

Tissue damage was significantly higher in the cyclophosphamide group than in the cyclophosphamide+coenzyme q10 group (p<0.05). The malondialdehyde levels were significantly higher and the activities of superoxide dismutase and catalase were lower in the cyclophosphamide group than in the cyclophosphamide+coenzyme q10 group (p<0.05).

**CONCLUSION:**

Coenzyme q10 may be a good option to prevent cyclophosphamide-induced kidney damage.

## INTRODUCTION

Cyclophosphamide is a nitrogen mustard-type alkylating agent. It has been utilized for the treatment of malignancies such as lymphoma, solid tumors, and autoimmune disorders^1^. It turns into phosphoramide mustard, which is its active metabolite, in the liver and gains effectiveness. Cyclophosphamide is hydroxylated in the liver and turns into a metabolite, acrolein, and side effects occur when acrolein is excreted by the kidney^2^. Numerous mechanisms could lead to kidney damage. Stankiewicz et al., reported that oxidative stress and elevated reactive oxygen species (ROS) could play an important role in cyclophosphamide-induced kidney damage^3^. Furthermore, prior studies demonstrated that cyclophosphamide could inhibit the activities of antioxidant enzymes such as superoxide dismutase (SOD) and catalase (CAT)^4,5^. Other theories related to nephrotoxicity due to cyclophosphamide were imbalance of the oxidants–antioxidants system, increase of the inflammatory cytokines, and apoptosis^6^, although there is no consensus that, to prevent the toxicity due to cyclophosphamide, a potent antioxidant agent could be useful.

Coenzyme q10 is a vitamin-like substance with antioxidant, anti-inflammatory, and anti-apoptotic activity, which is essential for the proper functioning of many organs and chemical reactions in the body, especially in the heart, liver, kidney, and pancreas^7^. Coenzyme q10 is in charge of the electron transport chain and controls redox reaction and metabolism^8^. Yousef et al., indicated that coenzyme q10 decreases ROS production and free radicals and reverses oxidative stress^9^. Therefore, it was thought that an antioxidant chemical such as coenzyme q10 might enhance the adverse effects of cyclophosphamide. In this study, we aimed to investigate whether coenzyme q10 has a protective effect against cyclophosphamide-induced damage to the kidney in rats.

## METHODS

Cyclophosphamide and coenzyme q10 were bought from a pharmacy (Kirsehir, Turkey). The study was reviewed and approved by the local ethical committee with the approval number 23/115 and date 07.06.2023. The study was carried out in Erciyes University Faculty of Medicine, Department of Histology and Embryology. A total of 30 female Wistar-Albino rats of 10–12 weeks old were included in the study. The animals were fed *ad libitum* feeding method with free access to water and food. All the rats were exposed to a temperature between 20 and 22°C under a 12-h light/12-h dark cycle.

### Study design

We planned an experimental animal study. In animal studies, groups are universally planned to be 6–8 rats on the basis of minimal animal use according to the 3R principle. The use of more rats is not ethically approved. When the literature is examined, it will be seen that animal studies are carried out according to this principle. In our study, we determined the number of animals in the study group according to these basic principles. As it is not possible to exceed 10 in a group due to the possible loss of rats, the sample size was planned to be 10 in each group.

A total of three groups were created. In group 1 (control group), neither any drugs were given nor anything was performed. In group 2 (cyclophosphamide group) (n=10), 30 mg/kg intraperitoneal cyclophosphamide was given for 7 days, and nothing was done. In group 3 (cyclophosphamide+coenzyme q10 group) (n=10), 30 mg/kg cyclophosphamide and 10 mg/kg coenzyme q10 were administered for 7 days intraperitoneally.

Anesthesia procedure was performed by utilizing ketamine hydrochloride (45 mg/kg, Ketalar, Eczacibasi, Istanbul, Turkey) and xylazine hydrochloride (5 mg/kg, Rompun, Bayer, Leverkusen, Germany). Blood samples were obtained from the animals by cardiac puncturing. The right kidney tissues were surgically extirpated. All rats were sacrificed via cervical dislocation.

### Biochemistry

Malondialdehyde (MDA) levels and SOD and CAT activities were measured by calculating absorbance in a spectrophotometer (Shimadzu UV 1800, Kyoto, Japan). The thiobarbituric acid test was used to calculate the MDA levels^10^. SOD enzyme activity was determined by Marklund et al. It was calculated according to the method reported by Marklund S and Marklund G^11^. CAT activity was measured as stated by Aebi et al^12^.

### Histopathological examination

Tissues were stored in 10% formaldehyde. Then, paraffin embedding was performed. The tissues were cut at 5 μm and stained with hematoxylin-eosin. Additionally, immunohistochemical p53 staining was performed. Histopathological assessment was performed by the same clinician via light microscopy (Olympus® Inc., Tokyo, Japan). The tissue damage was scored by determining the highest area. A modified semi-quantitative scoring was performed. Four categories were described (0: Absent, 1: Minimal, 2: Mild, 3: Moderate, and 4: Severe). Tubular dilatation, hemorrhage, necrosis, edema, inflammation, and glomerular atrophy were used to determine the degree of kidney damage. The histopathological assessment was performed according to the study reported by Neto et al^13^.

### Immunohistochemistry

p53 expression was graded using the 0–3+range (p53; 0: no staining, 1: less than 10% nuclear staining in renal tubular epithelial cells, 2: 10–30% nuclear staining, and 3: more than 30% nuclear staining).

### Statistical analysis

Statistical Package for the Social Sciences (22.00 SPSS Inc., Chicago, IL) was used for statistical analysis. Power analysis was used, and the sample size was calculated as at least 8 for each group with 80% accuracy. The chi-square test for categorical variables and the independent t-test for numerical values were used. A p<0.05 was considered statistically significant.

## RESULTS

Blood MDA levels and SOD and CAT enzyme activities are shown in Table 1. The MDA level was significantly higher in the cyclophosphamide group than in the cyclophosphamide+coenzyme q10 group (p<0.05). SOD and CAT activities were found to be significantly lower in the cyclophosphamide group than in the cyclophosphamide+coenzyme q10 group (p<0.05).

**Table 1:** Blood levels of malondialdehyde, superoxide dismutase, and catalase in serum samples of the groups.

	MDA (nmol/mg)	SOD (U/mg)	CAT (U/mg)
(Control group) (n=10)	16.59±7.08	29.4±10.81	69.58±17.72
(Cyclophosphamide group) (n=10)	35.12±13.85*	11.27±3.14*	27.56±10.41*
(Cyclophosphamide+coenzyme q10 group) (n=10)	23.66±10.27*	19.31±8.76*	40.67±16.70*

* Significant difference (p<0.05) between groups 2 and 3. Data are presented as mean±SD.

There was no difference between the groups in terms of the macroscopic appearance of the kidney tissue. Markers showing histopathological damage such as hemorrhage, edema, tubular dilatation, glomerular atrophy, and inflammation were more prominent in the cyclophosphamide group than the cyclophosphamide+coenzyme q10 group, and the differences were statistically significant (p<0.05) (Table 2).

**Table 2:** Distribution of histological damage according to the groups.

	(Control group) (n=10)	(Cyclophosphamide group) (n=10)	(Cyclophosphamide+coenzyme q10 group) (n=10)
Hemorrhage	0.00	2.00*	1.00*
Necrosis	0.00	1.00	1.00
Edema	0.00	2.00*	1.00*
Inflammation	0.00	2.00*	1.00*
Tubular dilatation	0.00	2.76*	1.56*
Glomerular atrophy	0.00	2.37*	1.03*

* Significant difference (p<0.05) between groups 2 and 3. Histopathological scoring was done by determining the highest area. Four categories (0: None, 1: Minimal, 2: Mild, 3: Moderate, and 4: Severe) were determined by making a semi-quantitative analysis, and the parameters were scored accordingly.

**Figure 1:**
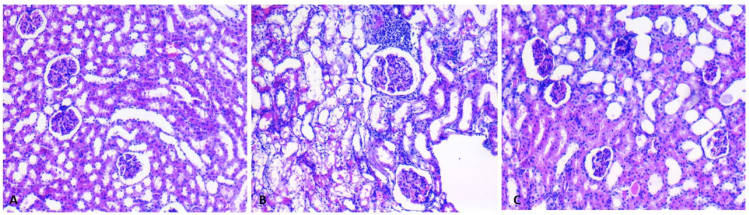
Demonstration of the histopathological examination by light microscopy. (A) View of renal parenchymal tissue from the control group (H&E, 200×). (B) Histopathological damage in the rats from the cyclophosphamide group. There was inflammation, minimal hemorrhage, and glomerular and tubular damage (H&E, 200×). (C) Renal parenchyma view of rats in the cyclophosphamide+coenzyme q10 group. Inflammation, hemorrhage, and tubulo-glomerular damage were improved (H&E, 200×).

When kidney tissues were evaluated microscopically, parenchyma structure, glomeruli, and tubules were normal in the control group (Figure 1A). In the cyclophosphamide group, hemorrhage, edema, inflammation, and glomerular and tubular injury were observed (Figure 1B). In the cyclophosphamide+coenzyme q10 group, it was observed that the damage in the renal parenchyma, tubular, and glomerular structures regressed (Figure 1C).

**Figure 2:**
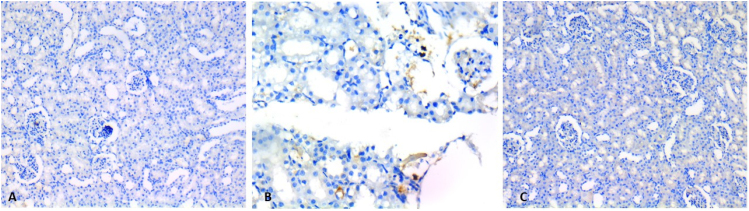
Evaluation of kidney with p53 immunostain. (A) Renal parenchyma view of rats in the control group (200×). (B) Renal parenchyma view of rats in the cyclophosphamide group. (200×). (C) Renal parenchyma view of rats in the cyclophosphamide+coenzyme q10 group (200×).

Sections made with the p53 immunostain were similar to the evaluations made with hematoxylin-eosin. It was observed that the histopathological damage, which was more prominent in the cyclophosphamide group, was reversed with the addition of coenzyme q 10 (Figures 2A–C).

## DISCUSSION

In this prospective randomized trial, we found significantly lower MDA levels and higher SOD and CAT enzyme activities in the cyclophosphamide+coenzyme q10 group than in the cyclophosphamide group. Also, tissue damage was common in the cyclophosphamide group and the addition of coenzyme q10 reversed the harmful effect of cyclophosphamide. We aimed to assess the effect of coenzyme q10 on cyclophosphamide-induced nephrotoxicity. To the best of our knowledge, this is the first experimental trial to investigate the protective effect of coenzyme q10 on renal toxicity due to cyclophosphamide.

Even though cyclophosphamide has been utilized in the treatment of malignancies, its toxicity due to cumulative dose is the main limiting factor^14^. Ahlmann et al., reported that, in addition to the gastrointestinal system, bone marrow, and cardiac toxicity, nephrotoxicity and hepatotoxicity can occur due to cyclophosphamide^15^. The main underlying mechanism of cyclophosphamide-induced kidney damage is oxidative stress. It leads to an increase in the levels of hydrogen peroxide, ROS, and hydroxyl radicals^16^. Antioxidant enzymes such as SOD and CAT tend to be lower in the rats given cyclophosphamide, and the addition of antioxidants such as amifostine reverses the whole picture and preserves the cell^17^. In our study, we observed that coenzyme q10, an antioxidant chemical, enhanced the biochemical and histological results in rats administered cyclophosphamide.

Coenzyme q10 acts through the benzoquinone ring in its structure and is involved in many reactions and processes in the cell, especially the electron transport chain^18^. It protects the cellular membranes from oxidative stress by reducing the levels of free radicals and ROS. Moreover, it also shows a direct antioxidant effect by increasing the effect of vitamins C and E^19,20^. It has been reported that coenzyme q10 levels are low in patients with chronic kidney disease. The addition of coenzyme q10 has been shown to improve kidney function and reduce the need for dialysis^21^. Kuang et al., demonstrated that SOD and CAT are major protective enzymes and prevent injury due to oxidative stress^22^. In our study, the tissue damage was more in the cyclophosphamide group than in other groups. Therefore, we assessed the effect of coenzyme q10 in rats given cyclophosphamide. In addition to biochemical and histological examinations, we strengthened our study by measuring the level of p53, which is closely related to oxidative stress and apoptosis. There are many publications in the literature showing the relationship between immunohistochemical expression and cell damage^13,23^. In these publications, it has been shown that p53 expression increases when cell damage increases after oxidative stress.

In our study, coenzyme q10 supplementation increased the activities of SOD and CAT and decreased the MDA levels. These findings were also confirmed histologically. The parameters demonstrating the histopathological damage such as tubular dilatation, hemorrhage, necrosis, edema, inflammation, and glomerular atrophy were significantly lower in the cyclophosphamide+coenzyme q10 group than in the cyclophosphamide group. In the examinations performed with p53 dye, it was shown that the addition of coenzyme q10 reduced the damage. Our negative aspects are that the findings obtained from the study are short-lived and the difficulty to adapt the data obtained from animal experiments to humans.

In conclusion, coenzyme q10 appears to be a promising and unique molecule in the prevention and treatment of cyclophosphamide-induced kidney injury.
